# Studying the Mechanism of Interaction of Doxofylline with Human Lysozyme: A Biophysical and In Silico Approach

**DOI:** 10.3390/molecules28083462

**Published:** 2023-04-14

**Authors:** Suliman Yousef Alomar

**Affiliations:** Zoology Department, College of Science, King Saud University, Riyadh 11451, Saudi Arabia; syalomar@ksu.edu.sa

**Keywords:** lysozyme, doxofylline, multi-spectroscopic, molecular docking, molecular simulation, drug–protein binding

## Abstract

In this study, multiple spectroscopic and computational methods were utilized to investigate the binding mechanism of doxofylline with lysozyme. The in vitro methods were used to obtain the binding kinetics and thermodynamics. UV–vis spectroscopy indicated the formation of complex between doxofylline and lysozyme. The Gibb’s free energy and binding constant from UV–vis data was obtained as −7.20 kcal M^−1^ and 1.929 × 10^5^ M^−1^, respectively. Doxofylline successfully quenched the fluorescence of lysozyme, confirming the formation of complex. The k_q_ and K_sv_ values for the quenching of lysozyme’s fluorescence by doxofylline were 5.74 × 10^11^ M^−1^ s^−1^ and 3.32 × 10^3^ M^−1^, respectively. These values signified a moderate binding affinity between doxofylline and lysozyme. In synchronous spectroscopy, red shifts were observed for indicating the changes in microenvironment of lysozyme following the binding of doxofylline. The secondary structural analysis was determined using circular dichroism (CD) which revealed an increase in % α-helical as a result of doxofylline interaction. The binding affinity and flexibility of lysozyme upon complexation have been revealed via molecular docking and molecular dynamic (MD) simulations, respectively. According to the many parameters of the MD simulation, the lysozyme–doxofylline complex was stable under physiological conditions. All during the simulation time, hydrogen bonds were continuously present. The MM-PBSA binding energy for lysozyme and doxofylline binding was found to be −30.55 kcal mol^−1^.

## 1. Introduction

Lysozyme, an innate immune system component that is also known as muramidase, N-acetylmuramide glycanhydrolase, or peptidoglycan N-acetylmuramoylhydrolase, is produced by animals [1]. With a molecular weight of 14.3 kDa and a wide distribution in cells and vertebrate secretions like sweat and tears [2], it is also found in egg white. In the pH range, the lysozyme enzyme is quite basic. The glycosidic bond formed between N-acetyl muramic acid and N-acetyl glucosamine is frequently regarded as a medical antibiotic because of its bactericidal capabilities, which induce this enzyme to hydrolyse the bacterial cell wall [3]. Lysozyme has been used for many years as a reference protein to study protein–ligand interactions due to its naturally high frequency [4]. Another crucial quality of lysozyme is its capacity to transport compounds that have physiological activity or therapeutic qualities. Lysozyme is a viable option for the medical and food industries due to its antibacterial qualities [5,6]. The lysozyme is known to exhibit certain pharmacological properties including antiviral properties and anti-histaminic properties [7,8]. Nonetheless, this protein is known to interact reversibly with small ligand molecules such as drugs and is used as a model protein to understand the mechanisms underlying the interactions of small molecules with the carrier proteins [9,10,11]. Considering such importance of lysozyme, this protein was selected for its interaction studies. 

Doxofylline is a bronchodilator that helps relax the smooth muscles of the airways in your lungs [12]. Asthma and other obstructive lung diseases are treated with it to either cure or prevent the problems associated with breathing. It is used to treat lung problem symptoms such wheezing, tightness in the chest, and shortness of breath [13]. This medication is available upon a doctor’s prescription in the form of a tablet, suspension, syrup, and injection. Doxofylline may cause adverse symptoms such as nausea, headaches, vomiting and stomach distress. Foods in which caffeine is present and beverages like coffee, tea, and dark chocolate are recommended not to be taken during the treatment period since they may increase the likelihood that side effects will occur [14].

Many scientists have investigated the interactions of numerous biological and synthetic compounds with the enzyme lysozyme over the years. Our research indicates that the precise doxofylline and lysozyme interaction has not yet been investigated. As a result, in this research, we have used UV-visible absorption and fluorescence spectroscopy to examine the complex formation. Through steady state fluorescence, the binding affinity and other aspects of binding were examined. Through 3D-Fluorescence and circular dichroic spectroscopy, it was possible to determine the microenvironmental and structural changes that happened in lysozyme following the binding of doxofylline. To further support the alterations in the microenvironment caused by lysozyme complexed with doxofylline, synchronous fluorescence was carried out. To verify the binding potential of doxofylline with lysozyme, in silico analysis techniques like molecular docking and molecular dynamic modelling were also used.

## 2. Results and Discussion

### 2.1. UV Absorption Spectroscopy Study

**Figure 1:**
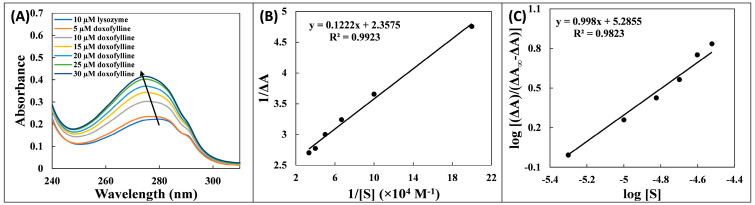
(**A**) UV absorption spectra of lysozyme (10 µM) titrated with different concentrations of doxofylline (0–30 µM). (**B**) plot of 1/(A − A_0_) vs. 1/[doxofylline]. And (**C**) Hill plot.

In several experimental settings, including protein–ligand binding, conformational modification has been evaluated using UV-visible absorption spectroscopy. Importantly, it offers insight into the induction of structural change and helps in the understanding of how drug and protein complexes arise [15]. Tyrosine, Tryptophan, and phenylalanine are the three aromatic amino acids that collectively absorb the most energy, contributing the most to the absorbance peak at 280 nm. The UV-visible spectra of lysozyme alone and in presence of doxofylline at increasing concentrations in phosphate buffer (pH 7.4) are shown in Figure 1A. The lysozyme absorption spectra increased as doxofylline was gradually introduced. The hyperchromicity of lysozyme’s spectrum indicates formation of complex with doxofylline along with the alterations in secondary form of the protein [16]. The blue shift that was also noticed may have been brought on by a decrease in polar environment surrounding the tryptophan and tyrosine residues along with increase in hydrophobicity. As doxofylline bonded essentially close to a tryptophan site, this indicated that the protein underwent a conformational change [17]. The alterations in absorbance spectrum of protein after the interaction of ligands is supposed to be due to the changes in secondary and tertiary structures [16]. The value of *K_d_* dissociation constant was calculated using Equation (3):

(1) $$\frac{1}{\Delta A}=\frac{K_{d}}{\Delta A_{\infty }\left[S\right]}+\frac{1}{\Delta A_{\infty }}$$

where ΔA = A − A_0_ is the difference in absorption of lysozyme–doxofylline complex and free lysozyme; [S] is doxofylline concentration; and ΔA_∞_ is absorbance difference at complete saturation of the protein. The plot in shown in Figure 1B, and *K_d_* value is listed in Table 1. The association constant (*K_a_*) was calculated from *K_d_* [*K_a_* = 1/*K_d_*]. Degree of cooperativity (*h*) was calculated using Hill Equation (4):

(2) $$\log\left[\frac{\Delta A}{\Delta A_{\infty }-\Delta A}\right]=h\log\left[S\right]+\log K_{a}$$

The values of ΔA_∞_ and log*K_a_* obtained from Equation (1) were used in Equation (2) for determination of *h*. The plot is depicted in Figure 1C, and the calculated *K_a_* value is 1.929 × 10^5^ M^−1^. The non-cooperative form of binding is indicated by the degree of cooperativity that is close to unity (0.98). Equation (5) was used to calculate the change in Gibb’s free energy (ΔG^0^):

(3) $$\Delta G^{0}=-RTlnK_{a}$$

where R represents universal gas constant; and *T* is temperature. The ΔG^0^ was obtained as −7.20 kcal M^−1^. The negative value of ΔG^0^ denotes the spontaneous character of the binding. A similar finding has been reported earlier where ΔG^0^ for the interaction of kaempferol with hen egg white lysozyme was found to be −6.63 kcal M^−1^. 

### 2.2. Steady-State Fluorescence Spectroscopic Analysis

**Figure 2:**
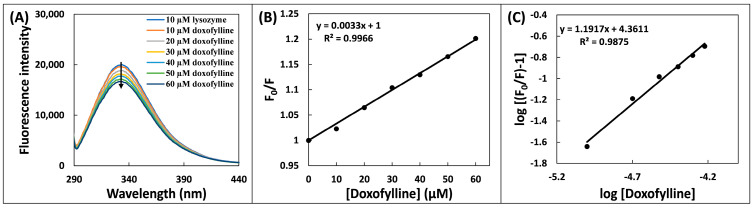
(**A**) Steady state fluorescence spectra of lysozyme (10 µM) in the absence and presence of varying concentrations of doxofylline (0–60 µM). (**B**) Stern–Volmer plot for the lysozyme and doxofylline interaction. (**C**) Double log plot for the lysozyme–doxofylline interaction.

Proteins are supposed to have inherent fluorescence because they contain amino acids, notably Trp and Tyr. The lysozyme emission peaks were at 341 nm when excited at 280 nm [18]. The fluorescence spectra of the lysozyme–doxofylline system are shown in Figure 2A, which demonstrates that when the excitation wavelength was 280 nm, the lysozyme fluorescence intensity consistently reduced with doxofylline addition, showing the potent binding of doxofylline to lysozyme. Moreover, the fluorescence quenching also indicates the alterations in the solvent accessibility of lysozyme in the vicinity of the protein following the binding of doxofylline [19]. Equation (6) and Equation (7) were used to derive the K_sv_ (Stern–Volmer constant) and k_q_ (molecular quenching constant rate) values from Stern–Volmer plot (Figure 2B) in order to explore the nature of quenching [20]:

(4) $$\frac{F_{0}}{F}=1+K_{sv}\left[Doxofylline\right]$$

(5) $$k_{q}=\frac{K_{sv}}{\tau_{0}}$$

where F_0_ and F are fluorescent signals of lysozyme and lysozyme–doxofylline complex; and τ_0_ is average lifetime of the fluorophore alone (5.78 × 10^−9^ s) [21]. The nature of quenching of protein’s fluorescence can be static or dynamic. Static quenching is generated by the development of ground state complex between quencher and fluorophore, whereas dynamic quenching is driven by collisions between the two [22]. However, with the mixed kind of quenching, both complex formation and fluorophore–quencher collision take place.

The values of k_q_ and K_sv_ were obtained as 5.74 × 10^11^ M^−1^ s^−1^ and 3.32 × 10^3^ M^−1^, respectively. Additionally, the lysozyme quenching operation commenced by doxofylline had rate constants k_q_ that were significantly larger compared to maximum scatter collision quenching constant of several quenchers whose value is typically 2 × 10^10^ M^−1^ s^−1^, indicating that quenching of lysozyme’s fluorescence by doxofylline was a static event [23].

The values of both i.e., binding constant (K_b_) and binding site’s number (n), were derived from the log plot (Figure 2C) using the Equation (8) [24]:

(6) $$log\frac{F_{0}-F}{F}=logK_{b}+nlog\left[Doxofylline\right]$$

Using the modified Stern–Volmer plot, K_b_ was obtained as 2.29 × 10^4^ M^−1^. The value of n was nearly one, denoting that there is one binding site for doxofylline in lysozyme.

### 2.3. Synchronous Fluorescence Examination 

**Figure 3:**
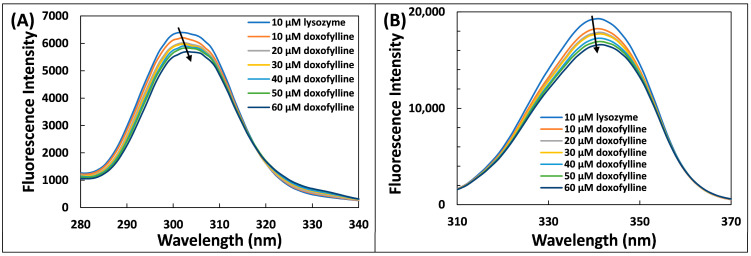
Synchronous fluorescence spectra for the interaction of lysozyme with doxofylline at (**A**) Δλ = 15 nm; (**B**) Δλ = 60.

Synchronous fluorescence is an important tool to study the changes in microenvironment around amino acids by analysing the shift in emission maxima, and it has certain advantages such as spectral simplification, sensitivity, and spectral bandwidth reduction [25]. By monitoring any potential shifts in emission maximum, it is possible to analyse the changes in polarity around the certain residues. Figure 3 shows synchronous fluorescence of Trp and Tyr residues. There is simultaneous excitation and emission of fluorescence signals where the difference between excitation wavelength and emission wavelength (Δλ) is kept constant. The changes in position of λ_max_ is the indicator of the changes in microenvironment around the chromophore [26]. By keeping the Δλ at 60 nm, it gives the changes in in microenvironment of tryptophan residue; while at Δλ = 15 nm, the changes around tyrosine residues are examined [23]. There was negligible shift of emission maxima at Δλ = 60 nm, indicating that there were negligible changes in microenvironment of tryptophan. However, the interaction of doxofylline with Lys altered the emission maxima peak with a redshift for Δλ = 15 nm, showing an increase in polarity around tyrosine and thereby lowering the hydrophobicity of it [27]. It is obvious from the data that interaction of doxofylline with Lys produced apparent changes in conformation of Lys [28].

### 2.4. Three Dimensional (3D)-Fluorescence Spectroscopic Study

Excitation-emission matrix spectroscopy (EEMS) is another name for it [29]. It was used to show how the microenvironment and conformation of lysozyme changed after it was bound to doxofylline. Table 2 lists the 3D-fluorescence peak values of lysozyme alone and lysozyme–doxofylline complexed (1:0 and 1:2) respectively. Peak a whose λ_ex_ and λ_em_ are 280 nm depicts Rayleigh scattering, while peak b whose λ_ex_ is 280 nm and λ_em_ is 540 nm is a second order scattering peak. Similarly, peak 1 with λ_ex_= 280 nm and λ_em_ = 340 nm and peak 2 with λ_ex_ = 230 nm and λ_em_ = 340 nm are due to the fluorescent nature of aromatic amino acids of the protein. The aggregation and alteration in the diameter of lysozyme may be responsible for the changes in scattering peaks, whilst the microenvironment and conformation in lysozyme are responsible for the changes in fluorescence peaks. The alteration of scattering peaks is linked to the change in lysozyme diameter modification in fluorescence peaks and is attributed to the conformational and microenvironmental perturbations of the proteins [30]. These findings of this experiment are also supplemented by the fluorescence quenching and synchronous fluorescence observations.

### 2.5. Circular Dichroism (CD) Measurements 

**Figure 4:**
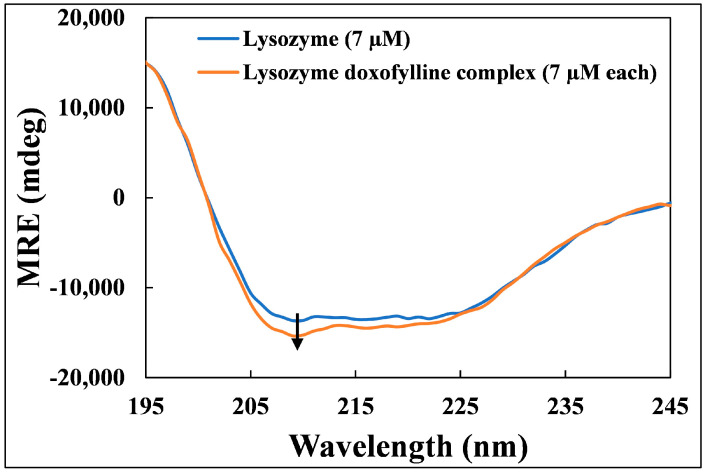
Far UV-CD spectra of lysozyme alone (7 µM) and lysozyme–doxofylline complex (7 µM each) at molar ratio of 1:1.

When a protein or enzyme interacts with tiny ligand molecules in an aqueous media, changes in its secondary structure are seen. These changes are studied using the circular dichroism (CD) approach. To evaluate changes in secondary structure of lysozyme after binding of doxofylline, we therefore used the Far-UV CD technique in this investigation. We looked at the spectral lines of lysozyme and lysozyme–doxofylline between 190 and 260 nm (Figure 4). Due to the lysozyme’s presence with the α -helical domain, there were two negative peaks (208 and 222 nm) which is attributed to the π–π* and n–π* transitions. The amount of α-helix in free lysozyme and lysozyme–doxofylline complex are listed in Table 3. The interaction of doxofylline with lysozyme slightly increased the α-helical content of protein. The data also supports that doxofylline stabilizes lysozyme. A contrary result was obtained for hen egg white lysozyme where the α-helical content decreased from 31.54% to 26.06% after the binding of triprolidine hydrochloride. The authors stated the interaction of triprolidine hydrochloride with hen egg white lysozyme resulted in destabilization of the protein’s secondary structure [31].

### 2.6. Molecular Docking Study

In silico molecular docking between lysozyme and doxofylline was performed in addition to spectroscopic experiments to further examine the binding location, mode, and energy in lysozyme–doxofylline interaction over course of time [32]. For docking, AutoDock Vina software was employed. The validation of docking methodology was performed by extracting the substrate molecule and then redocking it. It is interesting to note that the lysozyme substrate was docked at the same binding position as it was present earlier in the complex (Supplementary Figure S1). This validates the docking methodology. In the original lysozyme–substrate complex, the key residues involved in the binding were Asn46, Asn60, Tyr63, Trp64, Asp102, Gln104, and Ala108. 

**Figure 5:**
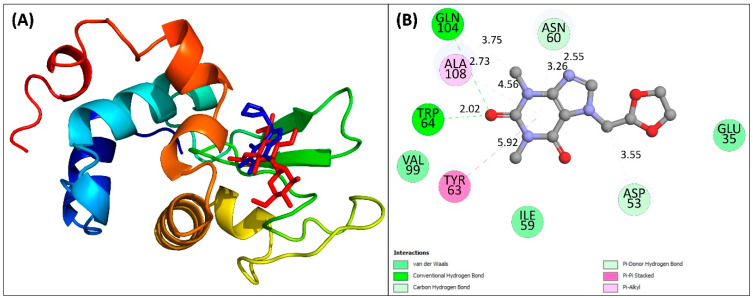
(**A**) Overlap of the docked structure of doxofylline with the original lysozyme–substrate complex. The doxofylline occupied same biding site as that of the substrate of lysozyme. Lysozyme is shown in ribbon model, doxofylline is shown as blue sticks, and substrate is shown as red sticks. (**B**) Two-dimensional view of the interaction of doxofylline with lysozyme.

In lysozyme–doxofylline docking, the structure with lowest energy was chosen for analysis in order to determine the binding characteristics. Doxofylline binds to lysozyme with a binding free energy of −6.5 kcal mol^−1^. It is interesting to note that doxofylline was docked at the binding site of its substrate (Figure 5A). Doxofylline formed two hydrogen bonds with Trp64 and Gln104 of lysozyme with bind length as 2.02 and 2.73 Å, respectively. Other residues such as Glu35, Asp53, Ile59, Asn60, and Val99 were involved in van der Waals forces (Figure 5B). Tyr63 and Ala108 interacted with doxofylline by hydrophobic interactions. It is obvious that the interaction involves both the residues of charged/polar and hydrophobic amino acids. Overall, the complex formation was also influenced by hydrogen bonds, van der Waals forces and hydrophobic interactions. Additionally, the complex’s stabilization also depends on the pi (π) interactions [33]. Certain residues including Asn46, Ile59, Asn60, Arg62, Tyr63, Trp64, Val99, Arg107, Ala108, Trp109, and Val110 are responsible for the binding of substrate to human lysozyme and catalyze the reaction. It is anticipated that the binding of doxofylline at the substrate binding site may lead to the inhibition in the enzymatic activity of this protein. 

### 2.7. Molecular Dynamic Simulation

**Figure 6:**
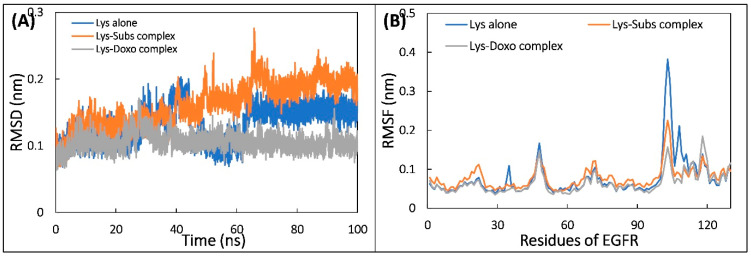
(**A**) The RMSD of backbone of lysozyme alone and lysozyme–substrate complex, and lysozyme–doxofylline complex during the simulation. (**B**) RMSF of Cα atoms of lysozyme alone and in presence of substrate and doxofylline.

The MD simulation was carried out in presence of physiological concentration of salt at 310 K to mimic the physiological conditions. The PBC corrections were done to the trajectories before their analysis. At first, analysis of the stability of trajectories was performed by calculating the RMSD (Figure 6A). The data shows that all systems were well equilibrated and trajectories did not show much deviations, showing that the systems were well stable [34]. The average RMSD of lysozyme alone, lysozyme–substrate complex, and lysozyme–doxofylline complex was 0.132, 0.161, and 0.104 nm, respectively. RMSF of C_α_-atoms of the systems were also calculated and data are shown in Figure 6B. The RMSF of most of residues were below 0.1 nm, which further confirms their stable nature [35]. The RMSF of residues from 100 to 110 in lysozyme–doxofylline complex remarkably reduced, which is due to the interaction of doxofylline that stabilized this region. The reduction in RMSF of this region was also observed in lysozyme–substrate complex. However, the fluctuations in residues of lysozyme were reduced to a greater extent in the presence of doxofylline compared to that of substrate alone. RMSF of each atom of doxofylline and substrate was also calculated (Supplementary Figure S2). The RMSF of both the ligands showed some variations, which is due to the motion of ligand at binding site. The atoms of substrate showed more fluctuations than the doxofylline, which may be due to more rotatable bonds present in the substrate [36].

**Figure 7:**
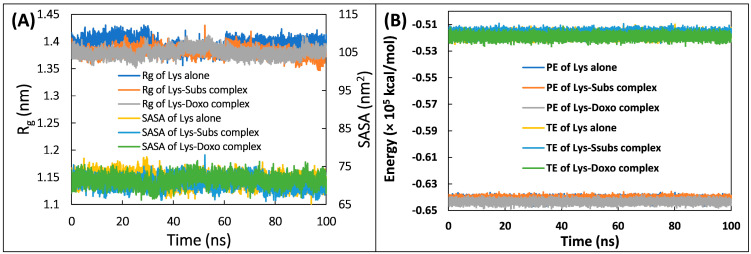
(**A**) Solvent accessible surface area (SASA) and radius of gyration (Rg) of lysozyme alone, lysozyme–substrate complex, and lysozyme–doxofylline complex as a function of simulation time. (**B**) Potential energy (PE) and total energy (TE) of lysozyme alone, lysozyme–substrate complex, and lysozyme–doxofylline complex as a function of simulation time.

The analysis of MD simulation data was further performed in which SASA (solvent accessible surface area, R_g_ (radius of gyration), and the energies were calculated. R_g_ is the mass-weighted RMS distance of the atoms from their common centre of mass. Analysing the changes in R_g_ over the course of simulation is considered an important indicator of the stability of proteins [37]. The R_g_ data is presented in Figure 7A. The R_g_ of all the systems were nearly identical throughout the simulation period, indicating their stability in aqueous environment, which also shows that the systems were stable and did not undergo any noticeable conformational changes during the simulation [38]. The average R_g_ of lysozyme alone, lysozyme–substrate complex, and lysozyme–doxofylline complex were obtained as 1.394, 1.380, and 1.3796 nm, respectively. A small decrease in the R_g_ of both complexes compared to lysozyme alone indicated that the protein got slightly more compacted following the complexation [39]. A similar data outcome was found for the SASA (Figure 7A). SASA is another critical indicator to examine the protein’s stability in MD simulation studies [40]. There were negligible changes in SASA of all three systems over time. Average SASA of lysozyme alone, lysozyme–substrate complex, and lysozyme–doxofylline complex were found to be 71.512, 70.586, and 71.426 nm^2^, respectively. The analysis of both R_g_ and SASA further confirmed the stable nature of lysozyme–doxofylline complex under physiological conditions [40]. Moreover, the energies of the trajectories were also calculated to further verify the stable nature of the systems (Figure 7B). Both the energies (total and potential) of all systems remained uniform throughout simulation, further confirming the stable nature of the systems. 

**Figure 8:**
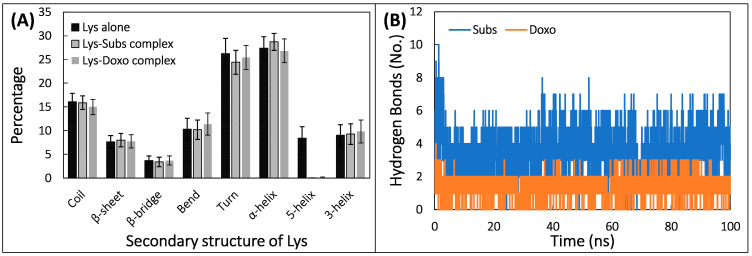
(**A**) Average percentage of secondary structure in lysozyme in the absence and presence of substrate and doxofylline. (**B**) Number of hydrogen bonds formed by substrate/doxofylline with lysozyme as a function of simulation time.

The impact of doxofylline’s binding on structure of lysozyme was examined by computing their secondary structures (Figure 8A). Average percentage of coils, β-sheets, β-bridges, bends, turns, α-helices, 5′-helices, and 3′-helices in lysozyme alone were found to be 14.99, 7.75, 3.71, 11.39, 25.45, 26.82, 0.01, and 9.84, respectively. The amount of α-helices and 7% β-sheets in huma lysozyme is consistent with the literature [41]. Likewise, average % of coils, β-sheets, β-bridges, bends, turns, α-helices, 5′-helices, and 3′-helices in lysozyme–doxofylline complex was 14.99, 7.75, 3.71, 11.39, 25.45, 26.82, 0.01, and 9.84, respectively. Similarly, average % of coils, β-sheets, β-bridges, bends, turns, α-helices, 5′-helices, and 3′-helices in lysozyme–substrate complex was 15.90, 8.01, 3.41, 10.20, 24.42, 28.73, 0.00, and 9.29, respectively. The negligible change in secondary structure of lysozyme in presence of doxofylline confirms the structural stability of complex in physiological conditions. The interaction of doxofylline with lysozyme was examined by analyzing the hydrogen bonds. Number of hydrogen bonds formed by substrate/doxofylline with lysozyme as a function of time is shown in Figure 8B. Average number of hydrogen bonds formed between doxofylline and lysozyme was 1.48. However, substrate formed greater number of average hydrogen bonds with lysozyme, which was 3.63. The hydrogen bond profile of the trajectory of complex was also examined which showed the continuous occurrence of hydrogen bonds in both the complexes throughout the simulation time. The highest percentage of hydrogen bond occupancy was shared by Trp64 and Asn60 in lysozyme–doxofylline complex. Similarly, these two residues (Trp64 and Asn60) also exhibited highest hydrogen bond occupancy for the complexation of substate to lysozyme. The data shows that doxofylline occupied the same binding site than that of the substrate. The competition of doxofylline for the same binding site may result in the inhibition of lysozyme’s enzymatic acidity. 

**Figure 9:**
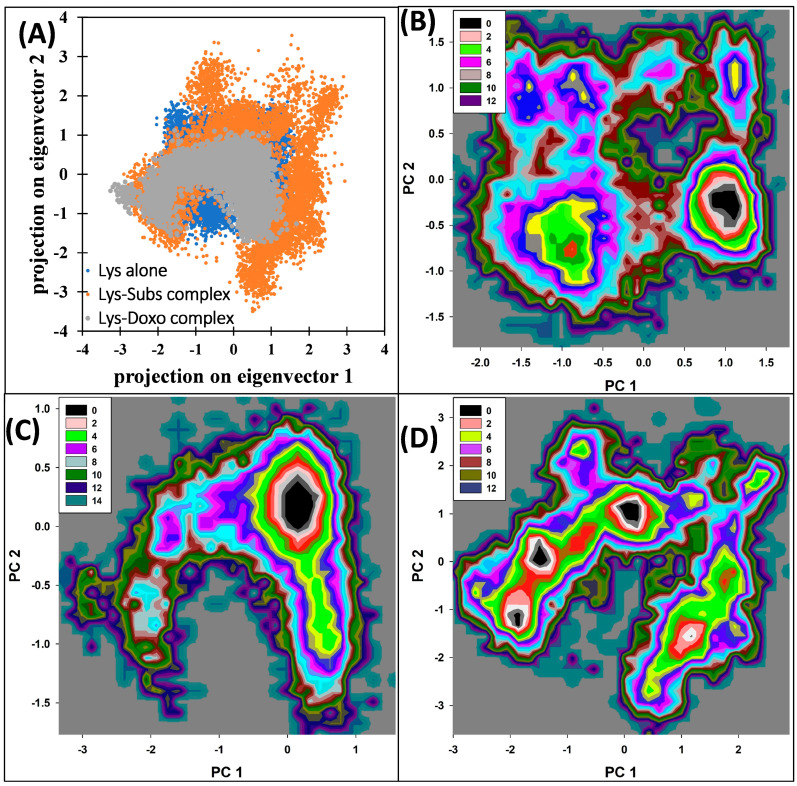
Principal component analysis: (**A**) Scatter plot of all trajectory frames of the backbone atoms of lysozyme alone, lysozyme–substrate complex, and lysozyme–doxofylline complex of the first two principal eigenvectors (PC1 and PC2). (**B**) Gibbs free energy landscape of lysozyme alone obtained from PCA analysis. (**C**) Gibbs free energy landscape of lysozyme–substrate complex obtained from PCA analysis. (**D**) Gibbs free energy landscape of lysozyme–doxofylline complex obtained from PCA analysis.

Principal component analysis (PCA) is a statistical method to examine a large set of data by reducing the dimensionality of data set without losing important information, which is called eigenvectors [42]. The analysis was done for studying flexibility in lysozyme both in uncomplexed and complexed form. The projection of eigenvectors is shown in Figure 9A. The 2D projection data shows that lysozyme–doxofylline complex and lysozyme alone occupies nearly the same conformational space. However, the conformational space occupied by lysozyme–substrate complex was slightly more than that of lysozyme alone. This shows that lysozyme–substrate complex was slightly more flexible in aqueous system compared to free lysozyme. The free energy landscapes were also calculated form the 2D projection data and the landscape is presented in Figure 9B–D. All systems reached the respective energy minima in their landscapes. In lysozyme alone and lysozyme–substrate complex, only one energy minima was found. However, in lysozyme–doxofylline complex, three energy minima points were found in the trajectory. The structures corresponding to the lowest energy were taken out for further examination using Ramachandran plots (Supplementary Figure S3). No residue was found to lie in the disallowed region of quadrant, further confirming the structural stability of lysozyme–doxofylline complex.

The role of various binding energies involved in binding of substrate and doxofylline with lysozyme was investigated using MM-PBSA analysis. One hundred frames were extracted from each trajectory at uniform intervals for MM-PBSA calculations. In typical protein–ligand interactions, non-covalent forces are the prominent one. These forces include van der Waals forces, hydrogen bonds, hydrophobic forces, and electrostatic forces. The forces either contribute positively or negatively to overall binding [17]. Various energies involved in binding of lysozyme with doxofylline are listed in Table 4. Overall binding energy for substrate and doxofylline were found to be −23.11 and −30.55 kcal mol^−1^, respectively. It is interesting to note that doxofylline exhibited higher biding energy than substrate. Their higher affinity of doxofylline towards binding site than substrate may be responsible for the inhibition of lysozyme. The energy contribution of each residue can also be calculated from the MM-PBSA data. The list of major energy contributors with their energy contribution is presented in Supplementary Table S1. In doxofylline–lysozyme interaction, Asn46, Asp49, Ile59, Asn60, Arg62, Tyr63, Trp64, Asp67, Val99, Arg107, Ala108, Trp109, and Val110 exhibited maximum energy contribution. Similarly, Asn46, Ile59, Asn60, Arg62, Tyr63, Trp64, Val99, Arg107, Ala108, Trp109, and Val110 were the key energy contributors in the substrate binding. Moreover, the key energy contributors were the same, further confirming the same binding site of substrate and doxofylline. 

## 3. Experimental Materials and Methods

### 3.1. Materials 

Human lysozyme and doxofylline were acquired from Sigma Aldrich, Bangalore, India. The remaining chemicals and solvents utilized throughout the entire study were of an analytical grade.

### 3.2. Sample Preparation

Doxofylline (2 mM) was dissolved in distilled water to make the stock solution. The sodium phosphate buffer (pH 7.4 and 10 mM) was used to prepare lysozyme stock solution of 0.5 mM. Doxofylline had a storage temperature of 4 °C, while lysozyme had a storage temperature of −20 °C. According to the needs, fresh working solutions were made using the dilution procedure.

### 3.3. Methods

#### 3.3.1. UV Absorption Spectroscopy Study 

Lysozyme (10 µM) was used to titrate different concentrations of doxofylline (0–30 µM), and UV absorption spectra were captured between 200 and 325 nm. The base line was corrected using the same buffer that was used to prepare the protein solution. First, the UV-vis absorption spectra (Shimadzu UV-1800, Kyoto, Japan) of lysozyme alone was recorded and then with the increasing concentrations of doxofylline. The data were used for the calculation of binding and thermodynamic parameters.

#### 3.3.2. Fluorescence Quenching Experiment 

The protein samples were excited at 280 for fluorescence quenching experiments. The protein’s fluorescent signal was recorded from 290 to 450 nm and the excitation and emission slit lengths were set to 5 nm each. The lysozyme concentration was fixed at 10 µM and concentration of doxofylline was varied from 0 to 60 µM. The highest fluorescence intensity points were used to calculate the quenching constant and Stern–Volmer constant. 

#### 3.3.3. Synchronous Fluorescence Examination 

In synchronous fluorescence studies, the concentration of lysozyme was kept at 10 µM and doxofylline was varied from 0 to 60 µM. To examine the changes in microenvironment of tyrosine, Δλ was set to 15 nm in which excitation of emission wavelengths was at 240 nm and 255 nm. Similarly, for tryptophan residues (Δλ = 60 nm), the excitation and emission wavelengths were fixed at 240 nm and 300 nm. 

#### 3.3.4. 3D (Three Dimensional) Fluorescence Emission Spectroscopic Study

Using Shimadzu RF-6000 (Kyoto, Japan) spectrofluorophotometer in 3D mode, the 3D-fluorescence emission spectrum of 10 µM lysozyme was recorded. The 3D fluorescence was also recorded in presence of varying ratios of doxofylline at 10 and 20 µM. The range of excitation wavelength was 200 nm to 400 nm. The emission wavelength range was from 200 nm to 550 nm. The scans were recorded at 6000 nm/min. 

#### 3.3.5. Circular Dichroism (CD) Measurements 

The CD spectra of lysozyme (7 µM) and lysozyme–doxofylline complex (1:1) were seen in the far-UV part of the spectrum between 190 and 260 nm. The speed of scan was 200 nm/min with data interval of 1 nm. The baseline spectrum of buffer alone (sodium phosphate buffer) was used to correct the obtained spectra of lysozyme and the complex. All CD measurements were performed at room temperature. The data presented is average of three replicates. The spectral data was used to calculate MRE (mean residue ellipticity) using Equation (1) [23]:

(7) $$MRE=\frac{Observed\;CD\left(mdeg\right)}{C_{p}\;nl\times 10}$$

where n is number of residues in lysozyme; l is cell’s path length; and C_p_ is lysozyme concentration. The % α-helix in lysozyme alone and in complex with doxofylline was calculated using Equation (2) [43]:

(8) $$\%\alpha -helical\;content=\frac{{(-MRE}_{208}-4000)}{33,000-4000}\times 100$$

where MRE208 is MRE at 208 nm; 4000 is MRE of random coil and β-form at 208 nm; and 33,000 is MRE of pure α-helix at 208 nm.

#### 3.3.6. Molecular Docking

First, the validation of docking procedure was performed. For the validation, the lysozyme complex containing its substrate (NAM-NAG) was taken. The substrate molecule was extracted and then redocked to check whether it binds to the same binding site. The substrate occupied the same binding site as it was present earlier, validating the docking procedure. 

For molecular docking, the coordinate of lysozyme (PDB ID: 1LZ1) was downloaded from RCSB PDB. The doxofylline structure was taken from PubChem (CID: 50942). The energy of protein structure was minimized using Swiss PDB viewer. The lysozyme structure was prepared in AutoDock Tools 1.5.6. The molecular docking was performed using AutoDock Vina [44]. For the preparation of receptor, the protein molecule was cleaned by removing the crystal water molecules and other molecules/atoms. Then, the polar hydrogen atoms were added, then Kollman charges were added. The receptor file was saved in pdbqt format. The size of grid box was 38 × 46 × 34 Å with the grid spacing of 1.00 Å. The center of the grid was x = 03.692, y = 19.019, z = 32.001. The structure of ligand (doxofylline) was also prepared in AutoDock Tools 1.5.6. The doxofylline molecule was made flexible by detecting the rotatable bonds to obtain the most energetically favorable conformation. The ligand file was also saved into pdbqt format. After docking, the confirmations were analyzed using PyMOL [45] and Discovery Studio [46]. 

#### 3.3.7. Molecular Dynamic Simulation

The docked complex was further taken for molecular dynamics (MD) simulation studies. Three simulations were run separately for lysozyme alone, lysozyme–substrate complex, and lysozyme–doxofylline complex. The lysozyme alone served as control. The MD simulation was performed using amber99sb-ILDN force field with the gromacs-2018.1 package [47,48]. The doxofylline and substrate topologies were generated using Antechamber packages of AmberTools21 [49]. All systems were solvated with TIP3P water model. The systems were then neutralized using the counter ions followed by their energy minimization to get rid of weak Van der Waals forces using steepest descent minimization. The minimized systems were first taken for NVT equilibration for 1 ns using the V-rescale thermostat [22]. Next, equilibration was done for NPT using the Parrinello–Rahman barostat at 1.0 bar for another 1 ns [50]. Both equilibrations were done at 310 K. The equilibrated systems were then used for one hundred nanoseconds MD simulation in which 10,000 frames of each trajectory were saved. All analyses were done using standard Gromacs-2018.1 utilities. The MM-PBSA calculations were done to calculate various binding energies and find out the major energy contributors [51]. 

## 4. Conclusions

The mechanism of doxofylline binding to lysozyme was elucidated in the current work using multiple spectroscopic and molecular docking techniques. Steady-state fluorescence and UV-absorption data verified the formation of complex. The binding constant for the interaction of doxofylline with lysozyme was in the range of 10^4^ to 10^5^ M^−1^. The fluorescence quenching was the result of a static process. The microenvironmental and structural changes in lysozyme on interaction with doxofylline were corroborated by 3D-fluorescence, circular dichroism, and synchronous fluorescence results. The values of binding energy provided by molecular docking techniques demonstrated that the binding interaction between lysozyme and doxofylline was in fact spontaneous and energetically favorable. Van der Waals interaction and hydrogen bonding were the principal forces at play. The doxofylline formed stable complex with lysozyme under physiological conditions. Our conclusions were further supported by in silico molecular dynamic simulation. Future research on the protein–drug interaction and the development of protein-specific therapeutics may benefit from the findings of this study.

## Figures and Tables

**Table 1:** Binding parameters for the interaction of doxofylline with lysozyme at 298 K obtained from UV–vis spectroscopy.

Temperature	*K_d_* (M)	*K_a_* (M^−1^)	*h*	ΔG^0^ (kcal mol^−1^)
298 K	5.18 × 10^−6^ M	19.29 × 10^4^ M^−1^	0.998	−7.20

**Table 2:** Excitation-emission peak values for the interaction of doxofylline with lysozyme obtained by 3D-fluorescence spectroscopy.

Lysozyme:Doxofylline	Peak 1 280/340	Peak 2 230/340	Peak a 280/280	Peak b 280/540
1:0	96,680.4	35,574.9	244,663.4	5036.2
1:2	96,008.5	34,751.4	229,976.1	4752.9

**Table 3:** α-helical contents and MRE values of lysozyme in the absence and presence of doxofylline estimated from the CD data.

Lysozyme Conc.	Doxofylline Conc.	Lysozyme:Doxofylline	MRE_208 nm_	% α-Helix
7	0	1:0	−13,273.95	31.97
7	7	1:1	−14,891.64	37.55

**Table 4:** Binding free energy of substrate and doxofylline with lysozyme calculated by the MM-PBSA method for 100 snapshots of MD simulation.

Energy Type	Ligand	
	Substrate	Doxofylline
ΔE_vdW_	−51.99 ± 0.33	−42.22 ± 0.37
ΔE_ele_	−38.60 ± 0.28	−61.84 ± 1.23
ΔE_PSE_	72.70 ± 0.48	78.62 ± 1.24
ΔES_SASA_	−5.25 ± 0.01	−5.12 ± 0.01
ΔE_BE_	−23.11 ± 0.47	−30.55 ± 0.56

ΔE_vdW_: van der Waal energy, ΔE_ele_: Electrostatic energy, ΔE_PSE_: Polar solvation energy, ΔE_SASA_: Solvent accessible surface area energy, ΔE_BE_: Binding energy.

## Data Availability

Data is contained within the article or Supplementary Material.

## Supplementary Materials

The following supporting information can be downloaded at: https://www.mdpi.com/article/10.3390/molecules28083462/s1, Figure S1: Overlap of the docked molecule of lysozyme substrate with the original complex. The substrate occupied same binding site with approximately similar orientation validating the docking procedure. The original substate is shown as blue sticks, the docked substrate is shown as magenta sticks, protein is shown as coloured surface view. Figure S2: Average RMSF of each atom of substrate and doxofylline. The RMSF is average of all frames of each trajectory. Figure S3: (A) Ramachandran plot of energy minima structure of free lysozyme alone. (B) Ramachandran plot of energy minima structure of lysozyme-substrate complex. (C) Ramachandran plot of energy minima structure of lysozyme-doxofylline complex. Table S1: Total binding energies (Etotal is total energy) (kcal mol^−1^) of major energy contributors for the interaction of substrate and doxofylline with lysozyme calculated from MM-PBSA.
