# Supplementary material for: Studying the Mechanism of Interaction of Doxofylline with Human Lysozyme: A Biophysical and In Silico Approach
Source: Molecules. 2023 Apr 14;28(8):3462. doi: 10.3390/molecules28083462 (PMC10146846; doi:10.3390/molecules28083462)
Supplement: Supplementary file 1 [file molecules-28-03462-s001.zip › molecules-2160270-supplementary.pdf]

Studying the mechanism of interaction of doxofylline with human lysozyme: A biophysical and in silico approach

Suliman Yousef Alomar

Zoology Department, College of Science, King Saud University, Riyadh-11451, Kingdom of Saudi Arabia.

Supplementary Figures

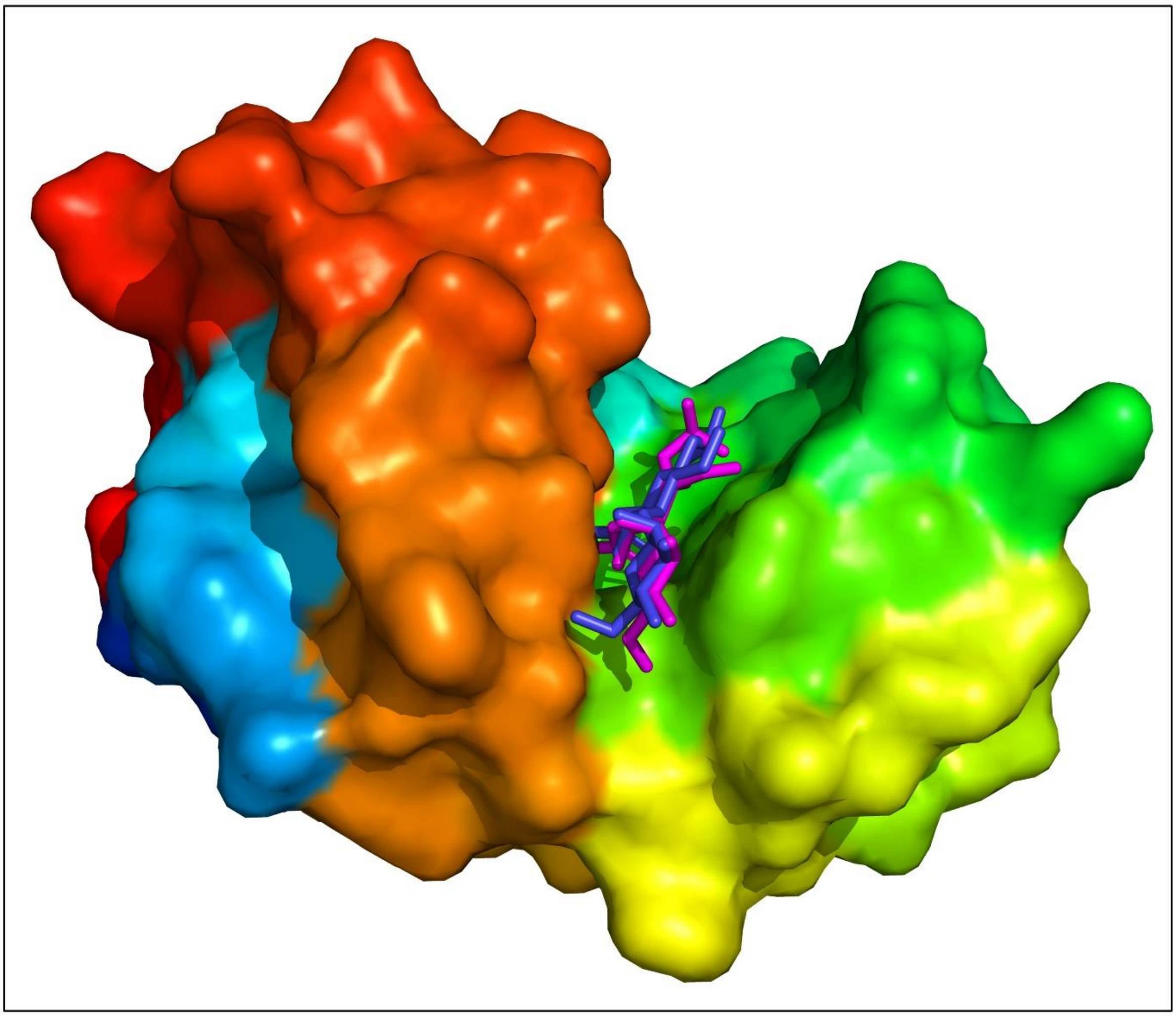

Supplementary Figure S1. Overlap of the docked molecule of lysozyme substrate with the original complex. The substrate occupied same binding site with approximately similar orientation validating the docking procedure. The original substate is shown as blue sticks, the docked substrate is shown as magenta sticks, protein is shown as coloured surface view.

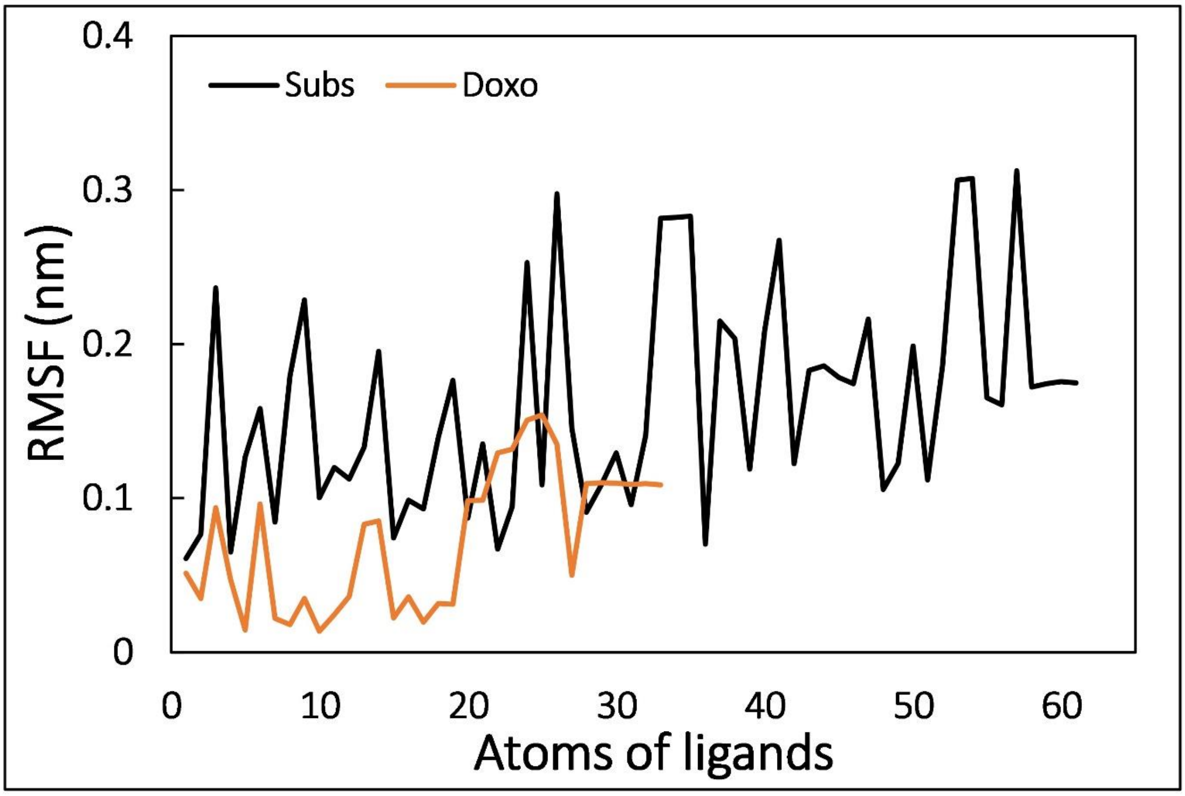

Supplementary Figure S2. Average RMSF of each atom of substrate and doxofylline. The RMSF is average of all frames of each trajectory.

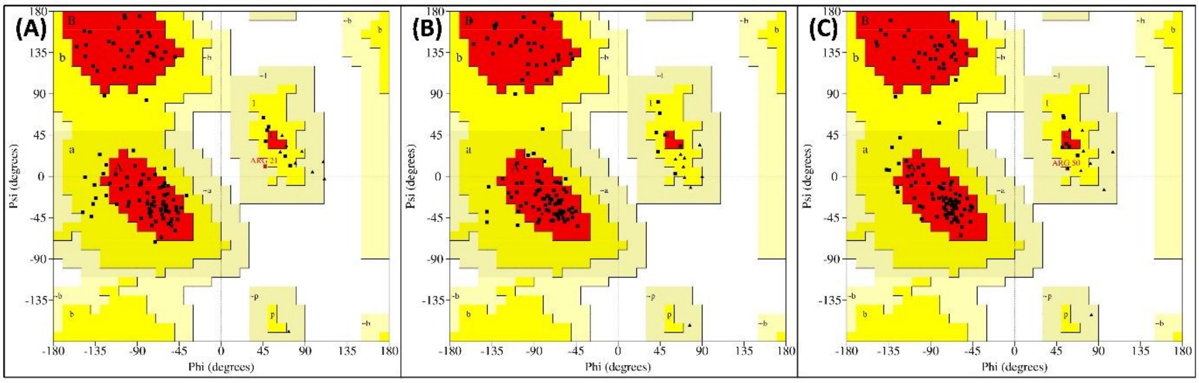

Supplementary Figure S3. (A) Ramachandran plot of energy minima structure of free lysozyme alone. (B) Ramachandran plot of energy minima structure of lysozyme-substrate complex. (C) Ramachandran plot of energy minima structure of lysozyme-doxofylline complex.

Supplementary Table

Supplementary Table S1. Total binding energies (E_{total} is total energy) (kcal mol^{-1}) of major energy contributors for the interaction of substrate and doxofylline with lysozyme calculated from MM-PBSA.

Substrate		Doxofylline	
**Residues**	**E_{total} (kcal mol^{-1})**	**Residues**	**E_{total} (kcal mol^{-1})**
Asn46	-0.236±0.021	Asn46	-0.129±0.005
Ile59	-0.581±0.045	Asp49	-0.137±0.030
Asn60	-1.564±0.092	Ile59	-0.205±0.021
Arg62	-0.422±0.044	Asn60	-0.922±0.027
Tyr63	-2.154±0.087	Arg62	-0.131±0.022
Trp64	-0.872±0.044	Tyr63	-2.385±0.049
Val99	-0.262±0.047	Trp64	-0.614±0.044
Arg107	-0.607±0.071	Asp67	-0.122±0.009
Ala108	-0.558±0.057	Val99	-0.464±0.022
Trp109	-0.818±0.070	Arg107	-0.307±0.021
Val110	-0.376±0.054	Ala108	-1.212±0.055
		Trp109	-1.255±0.044
		Val110	-0.111±0.012
